# Mean Platelet Volume as a Marker of Disease Severity in Hospitalized Children With Acute Bronchiolitis

**DOI:** 10.7759/cureus.115100

**Published:** 2026-08-24

**Authors:** Kedar Tilak, Trisha Sunderajan, Lewis Krata

**Affiliations:** 1 Neonatology and Pediatric Infectious Diseases, Children's Mercy Kansas City, Kansas City, USA; 2 Pediatrics, The Brooklyn Hospital Center, Brooklyn, USA

**Keywords:** acute bronchiolitis, acute respiratory infection, cbc testing, inpatient pediatrics, mean platelet volume(mpv)

## Abstract

Background: Bronchiolitis is one of the most common lower respiratory tract infections affecting children less than two years of age and is a leading cause of hospitalization. Mean platelet volume (MPV), a readily available component of the complete blood count, has been investigated as an inflammatory biomarker in various conditions. Our present study evaluated the association between MPV values and the severity of acute bronchiolitis in hospitalized children. Objective: The aim of this study was to determine the association between MPV, severity of respiratory distress, and need for oxygen support in hospitalized children under two years of age with acute bronchiolitis. Methods: A retrospective chart review was conducted of hospitalized children less than two years of age identified using International Classification of Diseases (ICD) codes for bronchiolitis. One hundred and fourteen charts met inclusion criteria. Data collected included age, length of hospital stay, degree of respiratory distress, respiratory support requirements, MPV values on admission, and respiratory syncytial virus status. Respiratory distress severity was assessed using a Clinical Respiratory Distress Score, which ranges from 1 to 12 and is categorized as mild (1-4), moderate (5-8), or severe (9-12). Chi-square tests were used to evaluate associations between MPV values, respiratory distress score, and need for oxygen support. Results: Low MPV was significantly associated with higher respiratory distress scores (moderate/severe) (χ²=4.93, p=0.026). Low MPV was also significantly associated with increased need for oxygen support (χ²=4.15, p=0.042). Conclusions: Low MPV at admission is associated with greater respiratory distress severity and increased oxygen support requirements in children hospitalized with acute bronchiolitis. MPV may have potential as an accessible marker of disease severity in children hospitalized with acute bronchiolitis.

## Introduction

Bronchiolitis is an acute viral infection of the lower respiratory tract that predominantly affects infants and young children and is the leading cause of hospitalization in children under two years of age [1,2]. In the United States alone, bronchiolitis accounts for about 100,000 hospitalizations annually at an estimated cost of $1.73 billion [3,4]. The disease is most commonly caused by respiratory syncytial virus (RSV), which is responsible for 60%-80% of cases, though other viruses such as parainfluenza, human metapneumovirus, and rhinovirus are also implicated in this disease [5]. The pathophysiology of bronchiolitis involves inflammation of the small airways, leading to edema of the bronchiolar wall, increased mucus production, necrosis and desquamation of ciliated epithelial cells, and accumulation of cellular debris [5,6]. These changes lead to progressive airway obstruction, gas trapping, atelectasis, and impaired gas exchange. The clinical presentation typically starts with predominantly upper respiratory symptoms, including rhinorrhea, cough, and low-grade fever, which later progresses to tachypnea, wheezing, increased work of breathing, and other signs of respiratory distress [5,6]. The management of bronchiolitis mostly includes supportive measures such as supplemental oxygen for hypoxemia, respiratory support, and adequate hydration [7,8]. Even though there has been extensive research conducted, no specific pharmacological intervention has demonstrated consistent efficacy in altering the disease course [7,9]. The American Academy of Pediatrics (AAP) guidelines recommend against the routine use of bronchodilators, corticosteroids, epinephrine, and antibiotics in the management of bronchiolitis [7,8]. Given the lack of disease-modifying therapies, it is important to identify patients at risk for severe disease who may require escalation of care. Presently, there is no universally accepted validated tool for predicting severe bronchiolitis [10]. There are many clinical severity scoring systems that have been developed, which include the Global Respiratory Severity Score (GRSS), the Bronchiolitis Severity Score (BSS), and the Respiratory Distress Assessment Instrument (RDAI), among others [11,12]. However, these scores mostly rely on clinical assessment parameters and fail to fully capture the underlying inflammatory burden of the disease. Mean platelet volume (MPV) is a routinely measured parameter on the complete blood count (CBC). It reflects platelet size and correlates with platelet activation and function [2,13]. MPV has recently emerged as a potential biomarker in various inflammatory and infectious conditions. Inflammatory mediators, particularly interleukin-6, can affect the megakaryocytic-platelet axis in the bone marrow, influencing platelet production and size [2,13]. While inflammation generally produces larger platelets with elevated MPV, paradoxically lower MPV values have been observed in certain acute and chronic inflammatory states [2,14]. This bidirectional response is thought to reflect the rapid consumption and turnover of large, activated platelets at sites of inflammation, which cause premature release of smaller platelets from the bone marrow [13]. Of relevance, Renshaw et al. demonstrated that RSV infection is strongly correlated with decreased MPV, with significantly lower MPV values in patients with positive versus negative RSV assays (9.7 ± 0.8 vs. 10.5 ± 0.9 fL, p<0.001) [1,15]. Similarly, decreased MPV has been observed in children with acute rotavirus gastroenteritis and in patients with active [5] tuberculosis compared with latent infection [2]. In contrast, elevated MPV has been reported in premature infants with respiratory distress syndrome and in children with multisystem inflammatory syndrome [2,16]. Given that CBCs are commonly performed in hospitalized patients, evaluating MPV as a marker of severity in bronchiolitis could provide a readily available, cost-effective tool to identify patients who may need a higher level of care earlier in their clinical course. The objective of this study was to determine the association between admission MPV values and the severity of acute bronchiolitis, as measured by respiratory distress scores and the need for oxygen support, in hospitalized children under two years of age. 

## Materials and methods

This was a single-center, retrospective observational cohort study using chart review conducted at a tertiary care community hospital in New York City over a two-year period from June 2020 to June 2022. Cases were identified through the medical records using International Classification of Diseases (ICD) diagnostic codes for acute bronchiolitis (J21.0, J21.1, J21.8, and J21.9). To minimize selection bias, we used consecutive (total-population, non-probability) sampling whereby all patients meeting the eligibility criteria during the study window were screened for inclusion. Eligible patients were children younger than two years (<24 months) admitted with a primary diagnosis of acute bronchiolitis who had a screening CBC with a documented MPV obtained on admission. Patients were excluded if they lacked an admission CBC/MPV value or had a high admission MPV (>12 fL). We excluded five patients with high MPV values because of their history of conditions associated with elevated MPV (chronic lung disease/bronchopulmonary dysplasia, congenital heart disease, and recent transfusions), since these can independently alter MPV or disease severity. Of the 119 charts initially identified, 5 were excluded for high MPV. Our final sample size was 114 patients. Age, comorbidities, and viral etiology (RSV status) were recognized as potential confounders that may independently influence both MPV and disease severity. Given the retrospective design and sample size, these variables were collected and described but not incorporated into a multivariable adjustment model. 

We de-identified all patient information and used a password-protected spreadsheet to include patients' age, length of hospital stay, admission MPV value, RSV status, degree of respiratory distress on admission, and the type or level of respiratory support required. The primary exposure, MPV, was classified using established laboratory reference ranges as low (≤7.5 fL), normal (7.6-12 fL), or high (>12 fL), with the analysis comparing low versus normal MPV. The ≤7.5 fL cutoff was based on the on-site clinical laboratory's own reference interval. The severity of respiratory distress on admission was quantified using a Clinical Respiratory Distress Score (range 1-12) [17] and categorized as mild (1-4), moderate (5-8), or severe (9-12), with moderate and severe combined into a single "moderate/severe" group for analysis, and the two outcomes of interest were respiratory distress severity (mild vs. moderate/severe) and the need for supplemental oxygen. Categorical variables were summarized as frequencies and percentages and continuous variables as mean ± standard deviation or median with range as appropriate. The association between MPV classification (low vs. normal) and each dichotomized outcome was evaluated using the Pearson chi-square test, with the Fisher exact test used where expected cell counts were <5, and effect sizes reported as odds ratios with 95% CIs where feasible. All tests were two-tailed; statistical significance was set at p<0.05; and analyses were performed using IBM SPSS Statistics software (IBM Corp., Armonk, NY). The study protocol was reviewed and approved by the Institutional Review Board/Ethics Committee and met the exempt criteria given the retrospective, minimal-risk design using de-identified data.

## Results

Of the 114 patients included, 32 (28.1%) had low MPV values (≤7.5 fL) and 82 (71.9%) had normal MPV values (7.6-12 fL). The distribution of MPV category across respiratory distress severity is presented in Table 1. Among patients with mild respiratory distress (n=75), 16 (21.3%) had low MPV and 59 (78.7%) had normal MPV, whereas among patients with moderate/severe respiratory distress (n=39), 16 (41.0%) had low MPV and 23 (59.0%) had normal MPV. Expressed by exposure, moderate/severe respiratory distress occurred in 16 of 32 patients with low MPV (50.0%) versus 23 of 82 patients with normal MPV (28.0%), yielding an absolute risk difference of 22.0 percentage points (95% CI, 2.1 to 41.8). Low MPV was associated with higher respiratory distress severity, with a crude odds ratio of 2.57 (95% CI, 1.10 to 5.96; Pearson χ²=4.93, df=1, p=0.026) (Table 1). The association between MPV category and the need for supplemental respiratory support is shown in Table 2. Among the 32 patients with low MPV, 22 (68.8%) required respiratory support, compared with 39 of 82 (47.6%) patients with normal MPV, an absolute risk difference of 21.2 percentage points (95% CI, 1.8 to 40.5). Low MPV was associated with an increased need for respiratory support, with a crude odds ratio of 2.43 (95% CI, 1.02 to 5.75; Pearson χ²=4.15, df=1, p=0.042) (Table 2). Taken together, the two analyses indicate that low admission MPV was associated with markers of greater disease severity, both in the graded severity score and in the objective requirement for respiratory support. These associations are unadjusted.

**Table 1:** Association between MPV category and respiratory distress severity in hospitalized children under two years of age with acute bronchiolitis Crude odds ratio for low MPV and moderate/severe severity, 2.57 (95% CI, 1.10 to 5.96). Absolute risk difference for moderate/severe distress (low vs. normal MPV), 22.0 percentage points (95% CI, 2.1 to 41.8). Association assessed using the Pearson chi-square test (χ²=4.93, df=1, p=0.026). A p-value <0.05 was considered statistically significant. fL, femtoliter; MPV, mean platelet volume; n, number of patients; %, row percentage within each severity category.

Respiratory distress severity	Low MPV (≤7.5 fL), n (%)	Normal MPV (7.6-12 fL), n (%)	Total, n
Mild	16 (21.3)	59 (78.7)	75
Moderate/severe	16 (41.0)	23 (59.0)	39
Total	32 (28.1)	82 (71.9)	114

**Table 2:** Association between MPV category and need for respiratory support in hospitalized children under two years of age with acute bronchiolitis Crude odds ratio for low MPV and need for respiratory support, 2.43 (95% CI, 1.02 to 5.75). Absolute risk difference for respiratory support need (low vs. normal MPV), 21.2 percentage points (95% CI, 1.8 to 40.5). Association assessed using the Pearson chi-square test (χ²=4.15, df=1, p=0.042). A p-value <0.05 was considered statistically significant. fL, femtoliter; MPV, mean platelet volume; n, number of patients; %, row percentage within each MPV category.

MPV	Respiratory support needed, n (%)	No respiratory support needed, n (%)	Total, n
Low MPV (≤7.5 fL)	22 (68.8)	10 (31.3)	32
Normal MPV (7.6-12 fL)	39 (47.6)	43 (52.4)	82
Total	61 (53.5)	53 (46.5)	114

## Discussion

This study demonstrated a statistically significant association between low admission MPV and both increased respiratory distress severity and a need for respiratory support in children under two years of age hospitalized with acute bronchiolitis. These findings suggest that MPV, a readily available and inexpensive laboratory parameter obtained from routine CBC analysis, may serve as a useful adjunct for identifying patients at risk for more severe bronchiolitis. However, the associations observed here are exploratory and require prospective confirmation. The findings of decreased MPV in more severely affected patients are consistent with prior observations in viral respiratory infections. Renshaw et al. reported that RSV infection is strongly correlated with decreased MPV, with significantly lower values in RSV-positive than RSV-negative patients [1]. The mechanism underlying this association most likely involves an inflammatory response to viral infections. During acute inflammation, many proinflammatory cytokines, such as IL-6, stimulate the production of thrombopoietin, which affects megakaryocytes, leading to increased platelet production [3]. Simultaneously, there is increased consumption of large activated platelets at sites of inflammation, with a compensatory release of immature, smaller platelets from the bone marrow, which causes a decrease in MPV [3]. In the context of bronchiolitis, where there is severe airway inflammation, we would expect greater platelet consumption and turnover, potentially explaining the association between lower MPV and more severe disease observed in this study. This pattern of decreased MPV in acute inflammatory states has been observed in other pediatric infectious conditions. Mete et al. demonstrated decreased MPV in children with acute rotavirus gastroenteritis compared with healthy controls [13]. Similarly, decreased MPV has been reported in patients with active tuberculosis during disease exacerbation [2]. The findings in these studies support the concept that MPV can serve as a negative acute-phase reactant in certain acute infectious and inflammatory conditions. The clinical significance of these findings lies in the potential utility of MPV as an early tool for triage. The management of bronchiolitis focuses on supportive measures, and current guidelines emphasize the importance of clinical assessment to determine the severity of the disease and the need for hospitalization and respiratory support [7-9]. There is no severity prediction tool that has been universally validated, and it is difficult to ascertain whether a patient is likely to deteriorate solely on the basis of clinical assessment [10]. The addition of assessing the MPV values to the clinical assessment could potentially provide objective, readily available data, which can support clinical decision-making regarding the level of care and the intensity of monitoring required. In our study, we saw that 68.8% of patients with low MPV required oxygen support compared with 47.6% of those with normal MPV, and 41.0% of patients with low MPV had moderate/severe respiratory distress compared with 21.3% of those with normal MPV. Although these showed statistical significance, we require further validation to use MPV as a standalone predictor. This includes determining optimal cut-off values, sensitivity, specificity, and predictive values. Several studies have examined platelet parameters in pediatric respiratory disease more broadly, providing useful context for the present findings. Sun et al. demonstrated that changes in platelet count during hospitalization for bronchiolitis were associated with disease severity, with decreasing platelet counts correlating with longer oxygen therapy duration and pediatric intensive care unit stay [4]. Gambadauro et al. analyzed the association between CBC parameters and novel inflammatory indices with bronchiolitis severity and demonstrated that lower age and weight at admission were most consistently associated with severe outcomes [3]. These studies, along with the present findings, suggest that platelet-related parameters may provide clinically useful information in the assessment of bronchiolitis severity. The present study extends this literature by focusing specifically on admission MPV as a dichotomized exposure and linking it to both a graded respiratory distress score and the objective need for oxygen support. Notably, in contrast to Renshaw et al., no association was observed in this cohort between RSV status and MPV, which may reflect differences in sample size, patient population, or the timing of sampling and warrants further investigation [1].

Our study had some limitations. To begin with, this was a retrospective study, which limited our evaluation of the patient in real time and adds a possible reporting bias. Secondly, our study did not control for potential confounders such as age of the patient, comorbidities, or viral etiology, which may independently influence both MPV values and disease severity. The Clinical Respiratory Distress Score used in this study, while providing a structured assessment, has not been independently validated in large multicenter studies. Finally, the study assessed only a single admission MPV value rather than serial measurements, so temporal changes in MPV during hospitalization could not be evaluated. Multivariable adjustment was not performed because of limited number of outcome events that restricts the number of covariates that can be reliably modeled, risking model overfitting and unstable estimates. Consequently, the reported associations should be interpreted as unadjusted, and residual confounding cannot be excluded.

## Conclusions

This study found that low admission MPV is significantly associated with greater respiratory distress severity and increased need for respiratory support in children less than two years of age who were hospitalized with acute bronchiolitis. These findings may point toward MPV being an easy, readily available, and cost-effective biomarker to help identify patients at risk for more severe diseases who may potentially benefit from closer monitoring and earlier escalation of care. However, in the absence of adjusted estimates, MPV should not be described as an independent predictor of disease severity. The present findings should be interpreted as hypothesis-generating associations that require confirmation in larger cohorts with a pre-specified multivariable analysis. Future prospective studies with larger sample sizes should aim to validate these findings, evaluate the independent predictive value of MPV after adjusting for confounders, and explore whether this association is stronger in RSV-associated bronchiolitis.

## References

[1] Renshaw AA, Drago B, Toraya N, Gould EW (2013). Respiratory syncytial virus infection is strongly correlated with decreased mean platelet volume. Int J Infect Dis.

[2] Guner Ozenen G, Sahbudak Bal Z, Umit Z (2021). Demographic, clinical, and laboratory features of COVID-19 in children: the role of mean platelet volume in predicting hospitalization and severity. J Med Virol.

[3] Gambadauro A, Mollica S, Rosa E (2025). Bronchiolitis severity affects blood count and inflammatory marker levels: a real-life experience. Viruses.

[4] Sun H, Xu H, Wang T (2020). The implications of platelet count changes during hospitalization in the disease management of paediatric patients with bronchiolitis. Infect Dis.

[5] Korniluk A, Koper-Lenkiewicz OM, Kamińska J, Kemona H, Dymicka-Piekarska V (2019). Mean platelet volume (MPV): new perspectives for an old marker in the course and prognosis of inflammatory conditions. Mediators Inflamm.

[6] Armarego M, Forde H, Wills K, Beggs SA (2024). High-flow nasal cannula therapy for infants with bronchiolitis. Cochrane Database Syst Rev.

[7] Chandelia S, Kumar D, Tandon D, Makol J (2026). Magnesium sulphate for treating acute bronchiolitis in children under two years of age. Cochrane Database Syst Rev.

[8] Zhang L, Mendoza-Sassi RA, Wainwright CE, Aregbesola A, Klassen TP (2023). Nebulised hypertonic saline solution for acute bronchiolitis in infants. Cochrane Database Syst Rev.

[9] Gill PJ, Anwar MR, Kornelsen E, Parkin P, Mahood Q, Mahant S (2021). Parenteral versus enteral fluid therapy for children hospitalised with bronchiolitis. Cochrane Database Syst Rev.

[10] Dalziel SR, Haskell L, O'Brien S (2022). Bronchiolitis. Lancet.

[11] Jat KR, Dsouza JM, Mathew JL (2022). Continuous positive airway pressure (CPAP) for acute bronchiolitis in children. Cochrane Database Syst Rev.

[12] Lee IR, Shin JI, Park SJ, Oh JY, Kim JH (2015). Mean platelet volume in young children with urinary tract infection. Sci Rep.

[13] Mete E, Akelma AZ, Cizmeci MN, Bozkaya D, Kanburoglu MK (2014). Decreased mean platelet volume in children with acute rotavirus gastroenteritis. Platelets.

[14] Erdede Ö, Sari E, Külcü NU, Sezer Yamanel RG (2023). The role of mean platelet volume in multisystem inflammatory syndrome in children with cardiac manifestations. Pediatr Infect Dis J.

[15] Sayed SZ, Mahmoud MM, Moness HM, Mousa SO (2020). Admission platelet count and indices as predictors of outcome in children with severe sepsis: a prospective hospital-based study. BMC Pediatr.

[16] Mazur NI, Caballero MT, Nunes MC (2024). Severe respiratory syncytial virus infection in children: burden, management, and emerging therapies. Lancet.

[17] Zaman S, Beardsley E, Crotwell D (2017). Bronchiolitis Pathway.

